# Implementing spiritual care education into the teaching of palliative medicine: an outcome evaluation

**DOI:** 10.1186/s12909-024-05415-0

**Published:** 2024-04-15

**Authors:** Yann-Nicolas Batzler, Nicola Stricker, Simone Bakus, Manuela Schallenburger, Jacqueline Schwartz, Martin Neukirchen

**Affiliations:** 1 Interdisciplinary Center for Palliative Care, University Hospital, Heinrich Heine University, Duesseldorf, Germany; 2 Evangelical Church in the Rhineland, Duesseldorf, Germany; 3 Institut Protestant de Théologie, Paris, France; 4 Evangelical Hospital Chaplaincy (Pastoral Care), University Hospital, Heinrich Heine University, Duesseldorf, Germany; 5 Department of Anesthesiology, University Hospital, Heinrich Heine University, Düsseldorf, Germany; 6 Interdisciplinary Centre for Palliative Medicine, Medical Faculty, Heinrich Heine University Duesseldorf, Moorenstr. 5, 40225 Düsseldorf, Germany

**Keywords:** Spirituality, Spiritual care, Palliative medicine, Education, Medical students

## Abstract

**Background:**

The concept of “total pain” plays an important role in palliative care; it means that pain is not solely experienced on a physical level, but also within a psychological, social and spiritual dimension. Understanding what spirituality entails, however, is a challenge for health care professionals, as is screening for the spiritual needs of patients.

**Objective:**

This is a novel, interprofessional approach in teaching undergraduate medical students about spiritual care in the format of a seminar. The aim of this study is to assess if an increase in knowledge about spiritual care in the clinical context is achievable with this format.

**Methods:**

In a mandatory seminar within the palliative care curriculum at our university, both a physician and a hospital chaplain teach strategies in symptom control from different perspectives (somatic domain – spiritual domain). For evaluation purposes of the content taught on the spiritual domain, we conducted a questionnaire consisting of two parts: specific outcome evaluation making use of the comparative self-assessment (CSA) gain and overall perception of the seminar using Likert scale.

**Results:**

In total, 52 students participated. Regarding specific outcome evaluation, the greatest gain was achieved in the ability to define total pain (84.8%) and in realizing its relevance in clinical settings (77.4%). The lowest, but still fairly high improvement was achieved in the ability to identify patients who might benefit from spiritual counselling (60.9%). The learning benefits were all significant as confirmed by confidence intervals. Overall, students were satisfied with the structure of the seminar. The content was delivered clearly and comprehensibly reaching a mean score of 4.3 on Likert scale (4 = agree). The content was perceived as overall relevant to the later work in medicine (mean 4.3). Most students do not opt for a seminar solely revolving around spiritual care (mean 2.6).

**Conclusions:**

We conclude that implementing spiritual care education following an interprofessional approach into existing medical curricula, e.g. palliative medicine, is feasible and well perceived among medical students. Students do not wish for a seminar which solely revolves around spiritual care but prefer a close link to clinical practice and strategies.

## Introduction

**Fig. 1:**
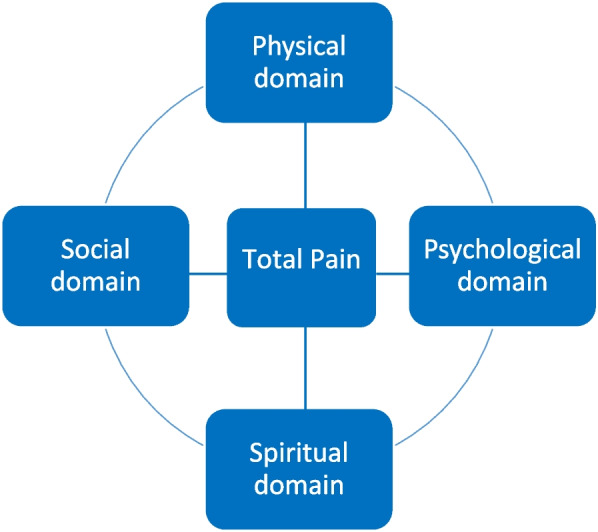
Total pain

Education in palliative care was introduced in 2009 as a compulsory subject in German medical curricula. In the 1960s, Dame Cicely Saunders established palliative medicine and hospices as we know them today. Back then, Cicely Saunders propagated the concept of “total pain”, which means that pain or suffering in general is not solely experienced on a physical level, but also within a psychological, social and spiritual dimension (see. Fig. 1) [1–4]. Understanding the importance of spirituality in everyday clinical practice and what it entails, however, is a challenge for health care professionals (HCP) in all medical disciplines across the world [5, 6]. Palliative care is a relatively young medical discipline which oftentimes is not sufficiently taught in medical curricula [1, 7] and, therefore, knowledge regarding the importance of spirituality, which at many faculties is integrated into palliative care education, is scarce [1, 7]. As a result, HCP tend to neglect the spiritual needs of patients [7, 8]. But, if there is no fundamental knowledge in regards of spirituality and spiritual care among physicians, how can they target total pain adequately?

The European Association of palliative care (EAPC) describes spirituality as following:

*“Spirituality is the dynamic dimension of human life that relates to the way persons (individual and community) experience, express and/or seek meaning, purpose and transcendence, and the way they connect to the moment, to self, to others, to nature, to the significant and/or the sacred.”* [1, 9].

It must be clear to all HCP that spirituality is a unique and subjective phenomenon that differs substantially from patient to patient [2, 10]. Furthermore, to fully address the spiritual needs of patients, self-reflection, thorough consideration of one’s own attitude towards death, and finding meaning in life, are essential [8, 9]. Several studies have shown the impact which the addressing of spiritual needs in the context of total pain can have on ameliorating the symptoms of patients, leading to a better quality of life and care [11–18]. Thus, once spiritual needs become imminent, it is necessary to engage in an interdisciplinary and multi-professional collaboration with specially trained professionals in the field of spiritual care [8, 10, 14, 15, 19]. Summing up, it is very important to raise awareness about the positive impact of spiritual care among HCPs [8, 15]. To increase such knowledge and accrue such skills, the teaching of spiritual care in medical curricula is essential [20]. Throughout different regions in the world, in-person didactic teaching on spiritual care is the most commonly used technique [5]. Usually, the teaching is based on case studies and many include screening strategies assessing spiritual needs [5]. Often, education on spirituality and spiritual care is part of curricula in palliative care [5, 21]. In German medical curricula, there is no compulsory subject solely revolving around spiritual care [22]. However, regarding the concept of total pain, implementing spiritual care into palliative care teaching, however, seems like a plausible proposition.

This study was conducted in order to assess the way medical students perceive the concept of implementing spiritual care into the teaching on symptom control in palliative care. Furthermore, we aimed to determine whether an actual increase of knowledge about spiritual care in the clinical context was achievable within this seminar.

## Material and methods

This study is a single-centre prospective study conducted at University Hospital Duesseldorf, Germany. Ethical approval was obtained by the local ethics committee (reference number 2022–2274).

### Curricular structure

At our facility, palliative care education is structured as followed: Five lectures (somatic symptoms, psychological symptoms, social symptoms and advance care planning, spiritual symptoms and end-of-life care and care for relatives, clinical ethics) and four seminars (symptom control, breaking bad news, clinical ethics I and II). Since 2022, the lecture on spiritual symptoms and end-of-life-care is held by both a physician and a hospital chaplain within the palliative care curriculum at Düsseldorf medical faculty. Beforehand, this lecture was solely held by a hospital chaplain. As internal evaluations implied, this concept was not well perceived by medical students as the relevance to daily clinical work was not apparent to them. They did not understand how spiritual care can support somatic strategies of symptom control and how both approaches are intertwined. Furthermore, they were unsure of how to assess patients’ spiritual needs. We therefore opted for the above-mentioned approach which allows lecturing relevant medical implications alongside spiritual care. As evaluations showed, this embeds spiritual care in a more clinical and tangible manner and students seem to better realize the relevance that spiritual care has in daily clinical practice. For example, students repeatedly stated that they were now able to understand the importance of ongoing collaborations for patients’ comfort care, e.g., in more sufficiently relieving anxiety or social distress.

Since this novel concept was perceived positively by medical students, we transposed it to our seminar titled “symptom control” which is now also held by a hospital chaplain and a physician. In the seminars, content from the lectures is further deepened and there is more room for discussions, e.g. concerning assessment of spiritual needs, possibilities of spiritual care, and inter-professional collaboration. There is also an emphasis on determining which patients might benefit from spiritual care making use of the SPIR tool (patient’s self-description as a *S*piritual person—*P*lace of spirituality in patient’s life – patient’s *I*ntegration in a spiritual community – *R*ole of health care professional in the domain of spirituality), which tackles different dimensions of spirituality [23].

**Fig. 2:**
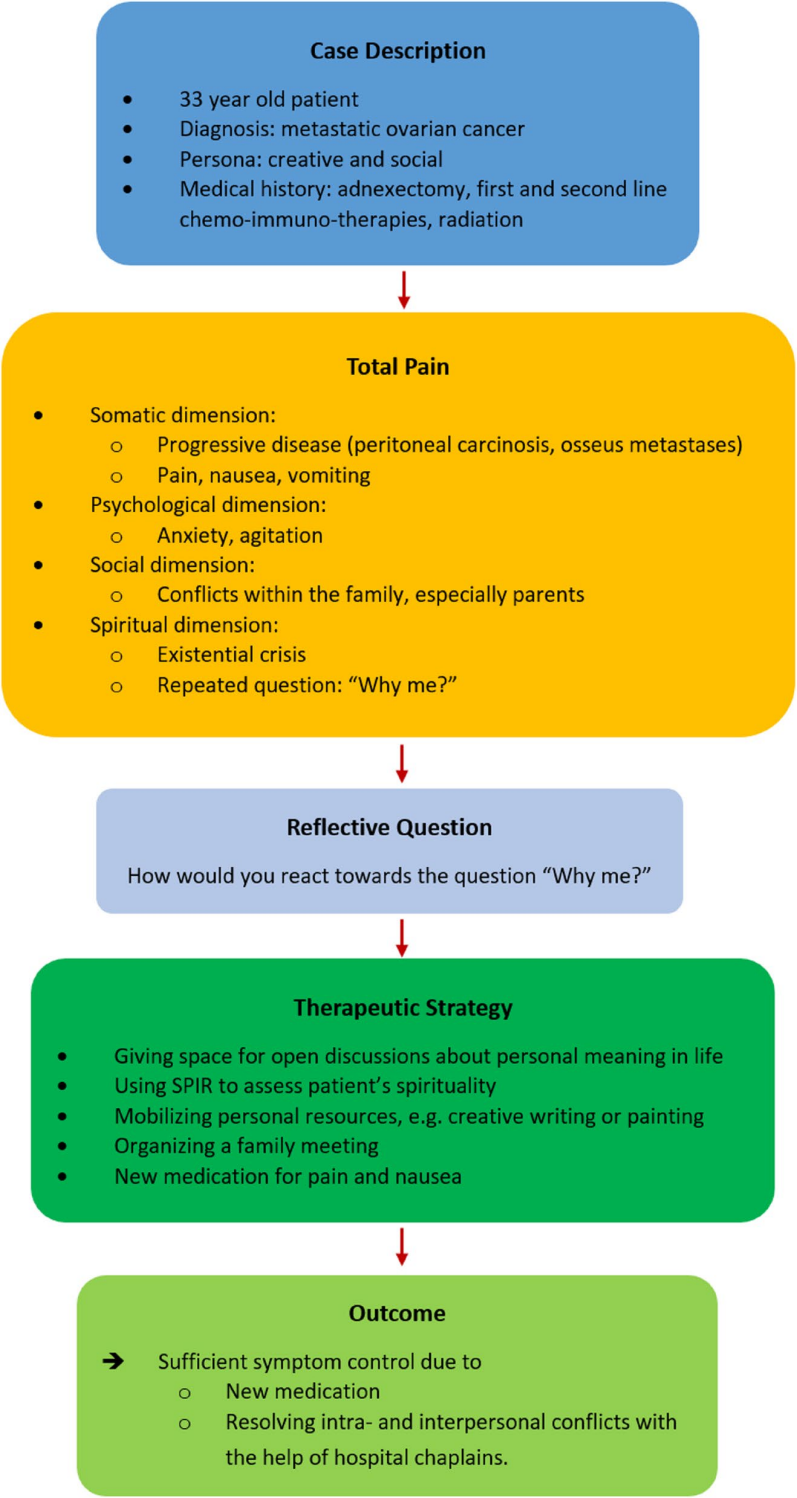
Case discussion in the seminar

In the seminar, a 33-year-old fictitious patient (inspired by a real patient) served as an example case. Her situation is used to address strategies for symptom control on both somatic and spiritual domains. To achieve this, a reflective question is discussed with the students followed by a joint development of possible therapeutic strategies on both the somatic and spiritual domain (see Fig. 2).

Our approach can be described as novel, since training in spiritual care often involves the mere shadowing of chaplains [5, 24–26]. An interprofessional, educational approach was mainly used with physicians or nurses in training [5, 27–29], but not with medical students.

### Evaluation methods

A structured, paper-based questionnaire was developed in repeated interdisciplinary and multi-professional discussions in the Interdisciplinary Centre for Palliative Care Medicine, University Hospital Düsseldorf, Germany. The basis for the questionnaire were the learning goals that are to be achieved within the seminar, as well as a didactic evaluation. The questionnaire was pretested among medical students, and unclear statements were altered. The questionnaire consists of two parts. The first part is made up of five statements regarding knowledge about total pain, assessing spiritual needs, and defining spiritual care (see Table 1) on both the knowledge and skills level. These statements cover the field of specific outcome evaluation. Making use of the comparative self-assessment (CSA) method to determine if a gain in knowledge was achieved, each student evaluated their knowledge before and after the seminar using the German school grading system (1 = “excellent” to 6 = “unsatisfactory”). The CSA gain is a well described and implemented method in evaluating actual knowledge gains in education [30–34]. This evaluation tool has the benefit of not taking into account experiences made beforehand as they are not contributing to the effect size [31]. CSA gain is calculated as followed:

$$\mathrm{CSA\, gain }\,\left(\mathrm{\%}\right)=\frac{\upmu \left({\text{pre}}\right)-\upmu \left({\text{post}}\right)}{\upmu \left({\text{pre}}\right)-1} \,x\, 100$$

**Table 1:** Statements

No.	Statement	Competency
1	I can define the concept of “Total Pain”.	Knowledge
2	I know about the relevance of “Total Pain”.	Knowledge
3	I know tools to assess patients’ spiritual needs in a structured pathway.	Knowledge
4	I can describe how spiritual care supports patients in everyday clinical practice.	Knowledge
5	I can identify patients who benefit from spiritual care.	Skills

Furthermore, CSA gain was calculated with a 95% confidence interval and standard error using individual learning gain (ILG) values. These values were calculated using the following formulas:

- ILG = 0 *if* pre = post and
- ILG = (pre − post)/(pre − 1) × 100 *if* pre > post [31].

The second part of the questionnaire consists of four questions regarding the perception of the seminar (structure, teaching spiritual care alongside symptom control in palliative care). A 5-Point-Likert scale was used for evaluation (1 = strongly disagree, 2 = disagree, 3 = neither, 4 = agree, 5 = strongly agree).

### Study participation and analysis

Participation in the study was anonymous, voluntary, and could be withdrawn at any time without explanation. Eligible participants were undergraduate medical students at the beginning of their fifth year of medical education (Germany: total of min. six years), who completed the mandatory palliative care course. The purpose and content of the study were presented orally, and, furthermore, written information and consent documents were handed out. After completion of the seminar, the questionnaire was handed out making use of a post-then design in which the students were asked to retrospectively rate their knowledge before and after the seminar. There were no exclusion criteria other than refusing to participate. Due to the small number of students per seminar (*n* = 15–20), no demographic characteristics besides sex were assessed.

Data analysis was performed using Microsoft Excel 2020 (version 16.42, Microsoft Corp., Redmond, WA, USA) and IBM SPSS Statistic version 28.0.1.1 (IBM, Armonk, NY, USA).

## Results

Throughout the course of one semester in 2023, the questionnaires were rolled out in each of six separate seminars. Out of 108 eligible attending students, 52 students participated in total (48.1%). 25% (*n* = 13) of the participants were of female, 75% (*n* = 39) of male sex. Within the answered questionnaires, there was no missing data.

**Fig. 3:**
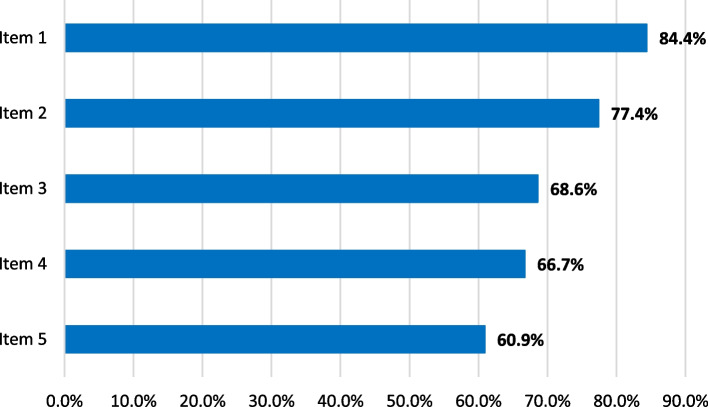
CSA gains for each item

Regarding the specific outcome evaluation, CSA gains showed a relevant increase especially in the field of knowledge (see Table 2 and Fig. 3). The greatest improvement (84.8%) was achieved in the ability of defining total pain and realizing its importance in clinical settings (77.4%). After the seminar, medical students were increasingly able to name tools such as SPIR in order to engage in spiritual needs assessment (CSA gain 68,8%). A lower increase in knowledge was achieved in realizing how spiritual care itself can benefit patients’ needs (66.7%). The lowest gain was detected in actually identifying patients who might benefit from spiritual care (60.9%), which represents a skill to be learned rather than knowledge to be gained.

**Table 2:** µ(pre), µ(post), corresponding CSA gains, 95% Confidence Interval (CI), and Standard Error (SE) for all five Items

Item	n	µ(pre)	µ(post)	CSA gain	95% CI	SE (%)
1	52	4.2	1.5	84.8%	0.86 – 0.94	0.020
2	52	4.1	1.7	77.4%	0.59 – 0.78	0.048
3	52	4.5	2.1	68.6%	0.60 – 0.74	0.072
4	52	3.7	1.9	66.7%	0.55 – 0.73	0.047
5	52	3.3	1.9	60.9%	0.46 – 0.66	0.053

Statistical analysis using 95% confidence intervals confirmed the gains in knowledge, which were significant for all items (Table 2).

**Fig. 4:**
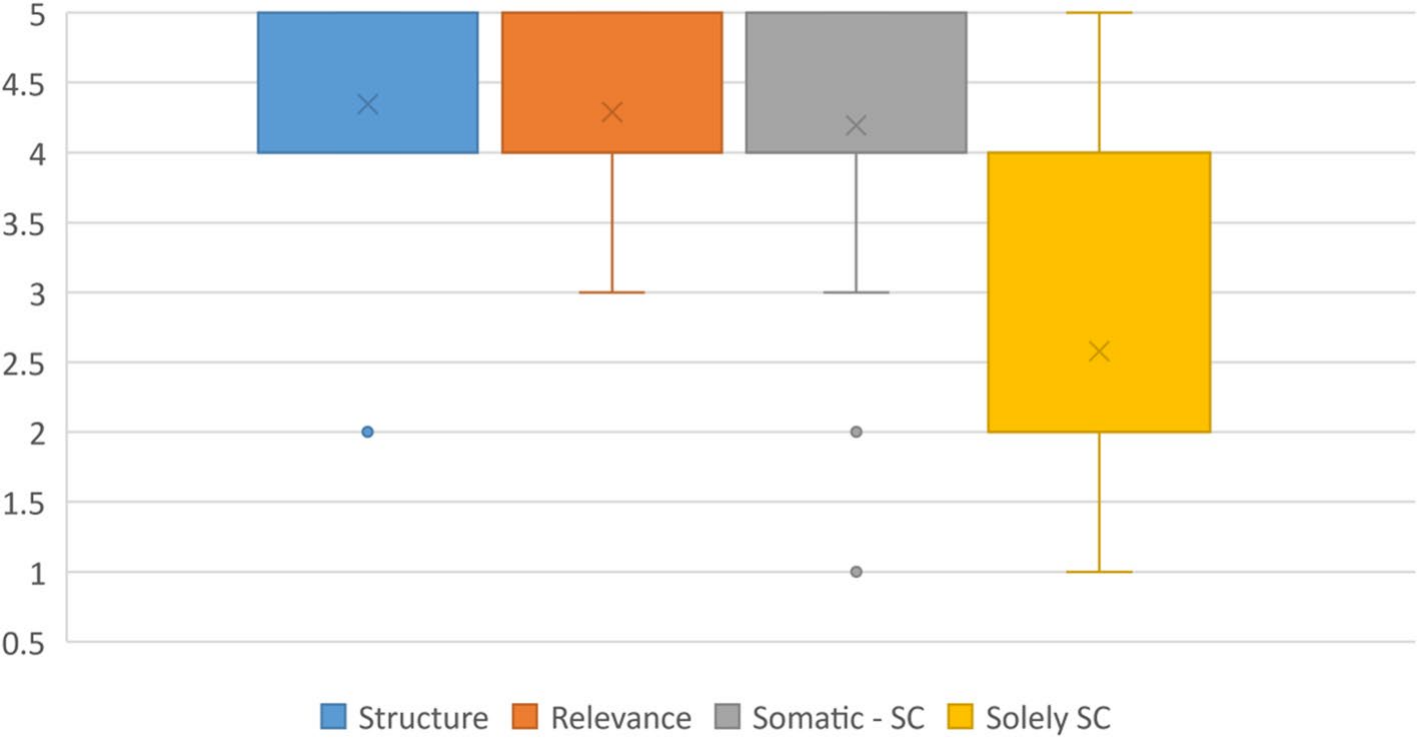
Perception of the seminar, Likert scale (1 = strongly disagree, 2 = disagree, 3 = neither, 4 = agree, 5 = strongly agree)

In regard to the second part of the questionnaire, students were overall satisfied with the new structure of the seminar (Table 3 and Fig. 4). The content was comprehensible and delivered clearly gaining a mean score of 4.3 (median 4, SD 0.6, min. 2, max. 5). The content was perceived as overall relevant to the later work in medicine (mean 4.3, median 4, SD 0.6, min. 3, max. 5). It seems as if medical students regard the implementation of spiritual care education into the seminar “symptom control”, which focuses on alleviating symptoms on multidimensional levels, as expedient. They feel that implementing education on spiritual care into this seminar makes sense (mean 4.2, median 4, SD 0.8, min. 1, max. 5). Furthermore, most students do not opt for a seminar solely revolving around spiritual care (mean 2.6, median 2, SD 1.3, min. 1, max. 5).

**Table 3:** Descriptive statistics for the perception of the seminar, Likert scale (1 = strongly disagree, 2 = disagree, 3 = neither, 4 = agree, 5 = strongly agree)

No.	Statement	mean	median	SD	min.	max.
1	The content is structured clearly and comprehensible.	4.3	4	0.6	2	5
2	The content is relevant to my later career.	4.3	4	0.6	3	5
3	Teaching spiritual care in a somatic seminar makes sense.	4.2	4	0.8	1	5
4	I prefer a seminar solely about spiritual care.	2.6	2	1.3	1	5

## Discussion

Our data show that implementing spiritual care education into existing medical curricula, in our example palliative care, is feasible and well perceived among medical students. The timing of our seminar is in accordance to other studies that found that spiritual care should be implemented in mandatory undergraduate courses [6]. Students do not wish for a seminar solely revolving around spiritual care but prefer a connection to clinical practice and strategies in symptom management. This enables them to understand the relevance of spiritual care in a daily clinical setting.

To evaluate training programs, Kirkpatrick proposed a four-level approach (level 1: reaction, level 2: learning, level 3: behaviour, level 4: results) [35]. We followed levels 1 (reaction—satisfaction) and 2 (learning—gains in knowledge) making use of the conducted questionnaire. Level 3 (change in behaviour – acquired skills) was briefly addressed with item 5 in the first part of the questionnaire. As level 4 is an indicator of direct results of the training at an organizational level, we were not able to incorporate items on this level. A different study among undergraduate nursing students assessed the effectiveness of teaching spiritual care in mandatory classes: There was an increase in knowledge, e.g., in defining spirituality, compared to students who obtained no information on spiritual care [36]. This is comparable to our study, as there were gains in knowledge after completing the mandatory seminar. We reached higher individual learning gains on the knowledge level than on the skills level, as was also the case in a number of other studies we conducted [31]. This is mainly because, due to the format of the seminar, no bedside teaching takes place and scenarios that might occur in everyday clinical practice can only be discussed and serve as examples.

The concept of total pain is essential in palliative care; however, it should not only be taken into consideration in a palliative setting, but whenever patients experience high burdens on various dimensions such as pain, anxiety, grief or existential distress [2, 4, 17, 37, 38]. We were able to thoroughly educate students on total pain and its relevance in clinical settings. Spirituality plays an important role in a holistic approach. However, literature shows that HCP often don’t know how to implement spiritual assessments and how to deal with spiritual needs [1, 5, 6, 8]. A systematic review on teaching methods found the usage of practical tools and the involvement of chaplains to be effective facilitators in the teaching of spiritual care [5]. A scoping review found that spiritual care should be taught in both mono- and multi-disciplinary educational settings [6]. With our multi-professional approach, we were able to introduce students to tools in assessing spiritual needs, such as SPIR [23]. Within this item, there was a definite gain in knowledge of these tools which make assessing spiritual needs of patients more feasible. This is in accordance with findings of a number other studies [5]. In our study, however, students are still unsure if they are fully able to determine which patients might actually benefit from spiritual care, even though this item still reached a learning gain of 60.9%. As concluded by other authors, there is need for ongoing education [5].

Even though our seminar entails many different aspects of the total pain concept (somatic symptom management, spirituality, and spiritual care) medical students found the content to be clearly structured and comprehensible. More importantly, they understood the relevance of spirituality for their future clinical work and perceived the multi-professional teaching as highly satisfactory. In sensitizing them in this, we hope that they keep in mind the importance of ongoing collaborations between different professions.

Our study has some limitations. Even though the questionnaire was pretested among medical students before the actual study, no validated questionnaire was used. The response rate of almost 50% is relatively low and it can be assumed that those who participated were mostly students who were interested in the topic. This might have led to bias as positive effects might have been overestimated. Due to the small study population and to protect the privacy of participating students, no demographic data besides sex was collected. Demographic data, however, might contribute to a better understanding of spirituality or palliative medicine beforehand such as age, professional expertise, or own spiritual resources. This also meant that adjusting for confounding factors was not possible. This study solely dealt with medical students and no patients were involved. It would be of interest to assess as to whether the content taught in this seminar ultimately impacts the wellbeing or stress levels of patients in everyday clinical practice. A study focusing on patients would complement the findings of this study, as suggested by other researchers [5]. Furthermore, the study was only performed in one centre; therefore, it can only serve as an example on how spiritual care education might be successfully implemented into medical curricula.

## Conclusions

Spirituality plays an important role for many people and should always be taken into consideration when treating patients. This especially applies to palliative care where the addressing of spiritual needs is of crucial importance [18]. However, many HCP don’t know how to address topics revolving around spirituality which makes it hard to determine which patients might benefit from spiritual care. Therefore, education on the nature of spiritual care, on what it entails and on how it can support patients in everyday clinical practice should be thoroughly integrated into medical curricula. We opted to implement spirituality and spiritual care into an existing seminar and lecture within the medical curriculum at our faculty. This was well received among students. As a result, we found a clear increase in knowledge about total pain and about the tools one might use to assess spiritual needs. This knowledge needs to be further strengthened in practical clinical scenarios.

## Data Availability

All data and materials are available within this publication.

## Abbreviations

HCP: Health care professional
EAPC: European Association of palliative care
SC: Spiritual care
